# e-Banking Adoption: An Opportunity for Customer Value Co-creation

**DOI:** 10.3389/fpsyg.2020.621248

**Published:** 2021-01-14

**Authors:** Rocío Carranza, Estrella Díaz, Carlos Sánchez-Camacho, David Martín-Consuegra

**Affiliations:** ^1^Department of Marketing, University of Castilla-La Mancha, Ciudad Real, Spain; ^2^Faculty of Management and Communication, Universidad Internacional de la Rioja, Logroño, Spain

**Keywords:** customer value co-creation, e-banking, e-services, technology acceptance model, PLS-SEM

## Abstract

The development of information and communication technologies offers innovative opportunities to establish business strategies focused on customer value co-creation. This situation is especially notable in the banking industry. e-Banking activities can support competitive advantages. However, the adoption of e-banking is not yet well-established among consumers. In this sense, the technology acceptance model (TAM) is considered essential in studying consumer behavior applied to adopt a particular technology. According to the TAM model, this study analyses the factors which influence bank customers to adopt e-banking to facilitate their banking services and support the process of value co-creation. Consequently, the authors examine five main aspects of the technology adoption model to provide a broad understanding of bank customers’ consumption of e-banking. A partial least squares structural equation modeling (PLS-SEM) analysis is conducted to evaluate proposed relationships between factors and customers’ e-banking adoption.

## Introduction

The rapid growth and development of information and communication technologies (ICT) have enabled companies to create value in a digital environment (Schreieck and Wiesche, 2017). Currently, the adoption of innovation in the organization’s strategy is an essential requirement to create value. The term value co-creation has a principal role in easing this innovation. O’Hern and Rindfleisch (2010) conceptualize value co-creation as a collaborative activity, in which consumers actively participate and choose components of a different product or service proposition. Thus, in the digital era, value creation has become the co-creation of value between customers and companies (Hosseini et al., 2020).

Internet and technological development have changed how financial services are offered and used (Malaquias and Hwang, 2019). Banks and many financial institutions suggest alternative innovative electronic channels for maintaining a competitive advantage and satisfying customer expectations. Mobile devices and destock have increasingly become tools that customers implement through e-banking to pay for products and services (Zhang et al., 2018). Therefore, e-banking can adapt to clients’ needs, such as performing banking activities, without physically visit an office or an ATM (Malaquias and Hwang, 2019). For this reason, e-banking has considerable value for many financial organizations and customers (Baabdullah et al., 2019).

The introduction and growth of Internet services, which offer better possibilities of interaction with companies, allow consumers to participate in the development and/or improvement of products/services, resulting in value. Consequently, organizations are concerned about attracting customers who want to contribute their ideas to the collaborative process (Chepurna and Criado, 2018). The banking context is particularly interesting in analyzing the transition toward a value co-creation strategy (Mostafa, 2020). The fierce competition in the banking arena has facilitated e-banking as the most cutting-edge electronic-based and self-service distribution channel (Malaquias and Hwang, 2019). e-Banking is conceptualized as a distribution and communication channel which allows customers to interact with a bank to conduct transactions economically and efficiently, mainly through electronic tools, e.g., tablets or smartphones (Singh and Srivastava, 2020). The use of e-banking offers a wide variety of services for customers, which provide them with value and create a competitive advantage over competitors, such as account checking, bill payment, transferences, or mobile phone text message notifications (Mostafa, 2020). As an example of this incremental service innovations, Bankia is modernizing their communication channels to increase the value offered to customers. Bankia has been recognized as the first Spanish bank with an official verified WhatsApp account to communicate with either current customers or prospects. This action is part of its business strategy “Digital Humanism” as a new way of relating to customers based on a closer, agile, and direct actions (Bankia, 2020).

The massive usage of the Internet and electronic gadgets have captured the attention of researchers to e-banking. Previous studies (e.g., Glavee-Geo et al., 2017; Singh and Srivastava, 2020) show that previous works have studied the factors that encourage the adoption of e-banking (Mostafa, 2020). However, the adoption rate of e-banking is below the expectation and still in the adoption phase, even though e-banking services offer several outstanding services to users (Shankar et al., 2020). Therefore, this study aims to develop an empirical model based on technology adoption, applied in e-banking to understand the behavior of the users. Specifically, some variables included in the technology acceptance model (TAM) will be examined as factors that stimulate the adoption of e-banking and become an opportunity for customer value co-creation.

For this reason, this research provides a series of contributions that can help identify decisive factors in the use of e-banking and encourage customer value co-creation through interaction with electronic services. In this setting, this study focuses on the following questions: What are the factors that affect a consumer’s use of e-banking? What factors are most important in the consumer’s intention to use e-banking? What type of e-banking is most in-demand, and what strategies around the use of e-banking could the banks and financial institutions follow to increase its use? How can the use of e-banking contribute to customer value co-creation? Through partial least squares structural equation modeling (PLS-SEM) approach and the use of the importance-performance map analysis (IPMA), this research field provides insights and recommendations to help the banking industry adopt and use e-services by consumers to support the process of value co-creation.

To achieve the proposed objective, the study is organized as follows. First, the conceptual framework, the proposed model, and its hypotheses are presented. Then, the methods used and the results of the study are described. Finally, the conclusions and limitations of the study are presented.

## Conceptual Framework

### Co-creation and the Banking Market

The banking industry is a leader in providing consumers with opportunities to access products and services through advanced technology (Malar et al., 2019). The development of ICT has allowed banks to have a relationship with customers, shifting away from physical interaction with a bank branch to interactive and virtual environments (Martovoy and Santos, 2012). Some authors, such as Andreu et al. (2010), specify the consequences of direct interactions between a company and its customers to achieve value co-creation. Other researchers, such as Payne et al. (2008), highlight that organizations must adopt a customer relationship approach to support value creation. Co-creation requires companies’ ability to connect with customers and market orientation to be closer to them (Ind and Coates, 2013). Consequently, the company-client relationship must be active, providing interactive experiences and activities guided by decisive practices while taking advantage of customers’ unconscious behavior. In this sense, customers are encouraged to participate in the process and meet their own needs.

Following the study of Grönroos (2011), consumers ought to perceive usefulness or benefit using self-service and involvement in the process to be motivated. In the banking sector, there is a generalized interest in providing easy and fast services, maintaining the quality of products, and services toward the customer. Furthermore, the advent of new technologies, products, and services encourages new needs and demands by customers (Hosseini et al., 2020). Ease access to information and the differentiation of products and services offered by the Internet creates higher expectations among customers. Consequently, an innovation that appears in a specific part of the work may be effortlessly accessed in other parts of the world and desired by any person (Mainardes et al., 2017). Another feature of electronic services is accessibility to consumers. Some studies indicate that banking services are linked to this new and demanding customer profile. Consequently, the new services provided by banks arise from customers’ needs, characterizing the continuous sharing of ideas and value co-creation in the banking sector (Oliveira and von Hippel, 2011; Akter et al., 2020).

Based on the study of Medberg and Heinonen (2014), direct contact with the company and e-services create new ways of relationship and involvement with customers, positively affecting the company’s financial performance (e.g., decreasing of operating costs, increase on investment return). Furthermore, this way of interacting with customers has boost competitiveness in the banking industry, requiring an agile adaptation from each financial organization. It is proven that, when a bank includes a new or enhanced service to customers, competitors follow this innovation through the launch of the same or improved service. Thus, co-creation characterizes the innovation and betterment of services provided by banks. This fact encourages customers’ active participation in the co-creation practice through several benefits: easer credit approval, lower charges, or commitment to the bank (Mostafa, 2020). Hence, value co-creation should drive to reciprocally favorable outcomes for both consumers and businesses.

### Adoption of Technology and e-Services Banking

In recent years, the development of Information Technology and the Internet has brought about changes in the performance of traditional services. Thus, e-banking has changed the conventional practices of banks and financial institutions and has captured the attention of both academics and practitioners (Wang et al., 2017). The adoption of e-banking is considered an innovative distribution channel for financial services due to rapid advances in e-banking applications and intense competence (Sikdar et al., 2015; Yaseen and El Qirem, 2018). Thus, understanding the adoption and use of e-banking has become a central research field. The literature indicates that the most relevant strength of the TAM, developed by Davis et al. (1989), is its generalizability and applicability in different contexts (Yaseen and El Qirem, 2018). This model is specifically indicated to study the intention to adopt specific technologies. Thus, the TAM applies models to study the acceptance and intention to use information system tools such as mobile commerce (e.g., Natarajan et al., 2018), m-banking (e.g., Mostafa, 2020; Shankar et al., 2020) and e-banking (Yoon and Steege, 2013; Salimon et al., 2017; Yaseen and El Qirem, 2018; Ahmad et al., 2019), among others. The original TAM considers perceived usefulness and perceived ease of use has a significant role in the technology acceptance process (Davis et al., 1989). On one side, perceived ease of use is defined as the degree to which a person believes that using a particular system is effortless, both physically and mentally. On the other side, perceived utility is described as the degree to which consumers believe that using a system will increase their performance (Davis et al., 1989; Mostafa, 2020). Some previous studies in technology acceptance demonstrate that perceived ease of use has a positive effect, mediated by perceived usefulness on the intention to use technology (Natarajan et al., 2018).

In the context of e-banking, it is observed that perceived usefulness represents one of the critical aspects that explain behavior intention to use e-banking (Malaquias and Hwang, 2019). For example, e-banking provides some unique services that are not available in offline banking, such as access to banking services at any time and from anywhere (Yoon and Steege, 2013; Shankar and Jebarajakirthy, 2019). Similarly, previous studies show the influence of perceived ease of using e-banking on perceived usefulness and attitude (e.g., Deb and Lomo-David, 2014). Internet and mobile technology should improve convenience for customers, and its ease of use is critical in customer usage. Some authors (e.g., Riquelme and Rios, 2010) claim that adopting mobile banking is influenced by consumer’s perceived ease of use due to a complex system when it performs financial transactions. In this sense, the authors highlight that if consumers perceive the performance of a financial transaction as easy through mobile devices, they will have a more favorable attitude toward adopting mobile banking (Zhang et al., 2018). Ahmad et al. (2019) argue that a client’s beliefs about the usability of the website or application affect his or her attitude toward the website or application. These authors state that the ease of use of e-banking systems is a critical factor in their adoption and evaluation by clients. Thus, the relationship between consumers’ attitudes toward the use of technology, an excellent example of this is e-banking, and perceived ease of use is studied (e.g., Zhang et al., 2018). Moreover, Mostafa (2020) argues that customers may negatively evaluate using e-banking if they believe e-banking technology is challenging to use and learn. Thus, the following hypotheses are proposed:

H1. Perceived ease of use positively influences on perceived usefulness of e-banking.

H2. Perceived ease of use positively influences on attitude toward using e-banking.

Another dimension included in the TAM model is the perceived usefulness. This concept and its role have been examined in e-banking works (e.g., Yoon and Steege, 2013; Salimon et al., 2017; Malaquias and Hwang, 2019). Perceived usefulness can be defined as a person’s belief about if the use of a specific technology will improve their task performance (Davis et al., 1989; Natarajan et al., 2018). Authors such as Yoon and Steege (2013) state that perceived utility is a positive and determining element in e-banking usage. Similarly, this term is the principal factor that impacts consumers’ attitudes toward the use of technology (Deb and Lomo-David, 2014). Consequently, customers will evaluate e-banking usage favorably if they perceive that e-banking has a relative advantage over other alternatives (Mostafa, 2020). Recently, authors such as Ahmad et al. (2019) have highlighted the positive relationship of perceived usefulness with both attitudes toward using e-banking and user intention. According to the previous statements, the following hypotheses are formulated:

H3. Perceived usefulness positively influences on attitude toward using e-banking.

H4. Perceived usefulness positively influences on intention to use e-banking.

The concept of attitude toward the behavior reflects the degree to which an individual assesses a specific behavior as useful or not (Ajzen, 1991). Venkatesh et al. (2003) interpret attitudes toward a specific innovation as results of an individual’s own beliefs about an objective and the evaluations associated with those beliefs. In TAM’s scope, positive attitudes toward innovative technologies have confirmed antecedents of intentions to adopt them (Davis et al., 1989; Schierz et al., 2010). The association among attitude and intention to use has been broadly examined in the literature, particularly in the banking literature (e.g., Shaikh and Karjaluoto, 2015; Zhang et al., 2018; Ahmad et al., 2019; Mostafa, 2020).

Similarly, past research shows that attitude is an essential determinant of behavioral intention and a relevant antecedent of actual behavior. Consequently, the intention to adopt has been analyzed to understand people’s actual behavior (Davis et al., 1989; Zhang et al., 2018). Yaseen and El Qirem (2018) conceptualize behavior intention to adopt e-banking services as a measure of the strength of an individual’s intention to perform a specific behavior. Also, authors such as Ahmad et al. (2019) explain behavioral intention to use e-banking as a precedent to the actual use of e-banking. Based on prior studies, the following hypotheses are proposed:

H5. Attitude toward using e-banking positively influences on intention to use e-banking.

H6. Intention to use e-banking positively influences on e-banking usage.

**FIGURE 1:**
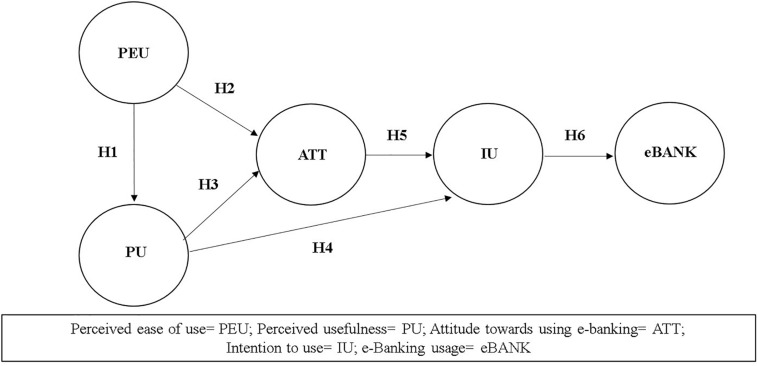
Model proposed on the e-banking usage with PLS-SEM.

Based on the above, Figure 1 summarizes the hypotheses of the proposed conceptual model.

## Materials and Methods

### Study Design

To test the proposed hypotheses, the authors carried out a study in Southern Europe’s banking industry. Specifically, the research was conducted in Spain due to the recent increase in e-banking in this country. e-Banking has experienced a growing acceptance in Spain in recent years, with more than 50% of digital banking population users. Some figures indicate that the number of Spain’s e-banking users increased to 28% between 2011 and 2019 (Statista, 2020a). Santander Group ranked first with more than 36 million digital customers during 2019, followed by BBVA with 31 million (Statista, 2020b).

A convenience sampling method was used to collect the data, taking e-banking users’ opinions as reference. A convenience sampling method was used to collect the data, taking e-banking users’ opinions as reference. Data was collected via an online survey from February to April 2020. Potential respondents in Spain were recruited through a national consumer panel. To measure each of the constructs, a self-administered survey has been used to analyze the e-banking usage of a set of well-known banks located in Spain. The application of PLS-SEM requires a minimum sample size. For this purpose, the statistical power is analyzed using G^∗^Power 3.1.9.7 (Carranza et al., 2020). Thus, the statistical power value for this sample considering a medium effect size (*f*^2^ = 0.15) is 0.989, higher than the established minimum of 0.8 (Cohen, 1988; Hair et al., 2019). Of 105 e-banking users (see Table 1), 45.7% of the sample collected is composed of men and 54.3% of women. Concerning age, the largest group is integrated by individuals between 24 and 33 years old, representing 32.4% of the sample. In addition, the accumulated percentage of consumers up to 43 years of age is 67.6%. Hence, the sample is predominantly made up of young adults and mid-aged e-banking users. Thus, this study coincides with previous studies in e-banking such as Zhang et al. (2018), Malaquias and Hwang (2019), Mostafa (2020), Singh and Srivastava (2020), where the samples are mostly composed of young people considered more likely to use digital technologies and media. Moreover, 35.3% of the respondents are employees, 43.8% are singles, and 37.1% are married. Concerning consumption factors, 93.3% of the sample uses e-banking to check their bank account balance, 49.5% make bank transfers through e-baking, and 15.2% manage invoices and taxes.

**TABLE 1:** Characteristics of the survey sample.

Customer profile	Categories	Sample (percentage)
Gender	Men	45.7
	Woman	54.3
Age	≥23	17.1
	24–33	32.4
	34–43	18.1
	44–53	14.3
	54–63	9.5
	+64	8.6
Occupation	Student	21.9
	Employed person	35.3
	Self-employed person	19.0
	Retired person	10.5
	Unemployed	11.4
	Other	1.9
Civil status	Single	43.8
	Married	37.1
	Living as a couple	10.5
	Separated or divorced	5.7
	Other	2.9

### Measures

In order to measure the constructs included in this study and examine the proposed relationships, a structured questionnaire was used. Firstly, questions related to the frequency and habits of the use of electronic banking were included. Then, the variables associated with the attitude and behavior toward using e-banking were exposed. All these constructs were evaluated with multi-item scales confirmed by previous studies, using a Likert scale ranging from 1 to 5, except the construct intended to use, presented on a semantic differential scale (see Table 2). Thus, variables for perceived ease of use were based on Davis et al. (1989) and Venkatesh et al. (2003). The attitude toward using e-banking was measured through a semantic differential scale using six items (five bipolar pairs of adjectives). Several authors, such as Stern and Salb (2015), define the attitude as a formative construct characterized mainly by affective aspects and instrumental distinctions. According to the scales proposed by Davis et al. (1989), Venkatesh et al. (2003), and Carranza et al. (2020) in the area of technology acceptance, the attitude variable was measured using three significant items (unpleasant-attractive, unsatisfactory-satisfactory, boring-fun) and three instrumental items (bad-good, uninteresting-appealing, harmful-beneficial).

**TABLE 2:** Measurement of key concepts.

Construct	Adapted items	Scale origin
Perceived ease of use (PEU)	PEU1: I find using e-banking easy.	Adaptation of Davis et al. (1989); Venkatesh et al. (2003)
	PEU2: I use e-banking without any help.	
	PEU3: I use e-banking without any problem.	
	PEU4: I consider myself an expert in the use of e-banking.	
	PEU5: Using e-banking does not require a tremendous mental effort.	
	PEU6: In general, I think it is easy to use e-banking.	
Perceived usefulness (PU)	PU1: Using e-banking enhances my effectiveness in living and working.	Adaptation of Agarwal and Karahanna (2000); Mostafa (2020)
	PU2: Using e-banking enhances my productivity.	
	PU3: I find e-banking useful in my living and working activities.	
	PU4: Using e-banking improves my performance in living and working.	
	PU5: In general, using e-banking is very useful.	
Attitude toward using e-banking (ATT)	ATT1: Bad – good	Adaptation of Davis et al. (1989); Venkatesh et al. (2003)
	ATT2: Uninteresting – appealing	
	ATT3: Harmful – beneficial	
	ATT4: Unpleasant – attractive	
	ATT5: Unsatisfactory – satisfactory	
	ATT6: Boring – fun	
Intention to use (IU)	IU1: Will you use e-banking in the next 3 months? 1. No, definitely not 2. Probably not 3. Indifferent 4. Yes, probably 5. Yes definitely	Adaptation of Bigné et al. (2008)
e-Banking usage (eBANK)	eBANK1: I have no problem with the use of e-banking.	Adaptation of Davis et al. (1989); Dutot (2015)
	eBANK2: No one influences my decision to use e-banking.	
	eBANK3: I have enough experience with new technologies to use e-banking.	

The perceived usefulness was measured using the Agarwal and Karahanna (2000) scale, following Mostafa’s (2020) work. Intention to use was measured using a single-item scale based on previous research, such as Bigné et al. (2008). Three items adapted from Davis et al. (1989) and Dutot (2015) were used to measure e-banking usage. The last section of the questionnaire aims to collect information on the socio-demographic profile of e-banking users, such as gender, age, or occupation.

### Statistical Analysis

The model was estimated using PLS-SEM. PLS-SEM is a technique of structural equation models based on variance. In this study, the use of PLS-SEM is recommended because (1) the study includes a formative construct (attitude toward using e-banking), (2) the model uses composite models (Hair et al., 2019), and (3) PLS-SEM is applied in recent studies of TAM, in the field of e-banking, as well as in other different areas (e.g., Salimon et al., 2017; Carranza et al., 2020; Zollo et al., 2020). To estimate the proposed model, SmartPLS 3.2.9 was used. According to Hair et al. (2019), a two-stage approach is used to evaluate the proposed model in this e-banking customers’ context. Thus, the measurement model is evaluated distinguishing the variables considered as a composite model in Mode A and Mode B, and then, the structural model is assessed.

## Results

### Measurement Model

First, the standardized root mean square residual (SRMR) of the proposed model is calculated in order to assess the model fit (Henseler et al., 2016). In this case, the SRMR value is 0.070, indicate an appropriate fit, given the accepted 0.008 cut-off point. To evaluate the measurement model, the reliability of the scales is studied for the construct’s perceived ease of use, perceived usefulness, intention to use, and e-banking usage (Mode A). Thus, the loadings of the indicators are examined, all of which are higher than 0.708. The evaluation of individual reliability is examined through the Dijkstra–Henseler’s rho (ρ_*A*_) and the composite reliability (CR) being higher than 0.7 in all cases (Hair et al., 2019). Therefore, all the variables included in the model reflect high internal consistency (see Table 3). Then, the average variance extracted (AVE) is used to evaluate convergent validity. In this case, all values of the AVE are within the established thresholds limits (Fornell and Larcker, 1981). Lastly, all loadings are significant at 99.9% (Hair et al., 2017). Concerning the analysis of the discriminant validity, the results obtained by the Fornell–Larcker criterion show a satisfactory degree of discriminant validity. However, Henseler et al. (2015) suggest construct thresholds below 0.9 for HTMT to establish discriminant validity. In this case, problems of discriminant validity between PEU and PU are detected. For that reason, the items causing the problem are studied and eliminated (see Table 4).

**TABLE 3:** Measurement model evaluation.

Construct/associated items	Loading	Dijkstra–Henseler’s rho (ρ_*A*_)	CR	AVE
PEU (Mode A)				
PEU2	0.924***	0.906	0.937	0.831
PEU4	0.912***			
PEU5	0.899***			
PU (Mode A)				
PU1	0.916***	0.900	0.936	0.831
PU3	0.923***			
PU4	0.895***			
IU (Mode A)				
IU1	1.000***	1.000	1.000	1.000
eBANK (Mode A)				
eBANK1	0.918***	0.934	0.926	0.807
eBANK2	0.852***			
eBANK3	0.923***			

*n* = 5,000 subsample: ***p < 0.001 (one-tailed *t* Student). CR, composite reliability; AVE, average variance extracted; PEU, perceived ease of use; PU, perceived usefulness; IU, intention to use; eBANK, e-banking usage.

**TABLE 4:** Measurement model: discriminant validity.

	Fornell–Larcker criterion					HTMT			
	EU	PU	IU	eBANK		PEU	PU	IU	eBANK
PEU	**0.912**				EU				
PU	0.811	**0.912**			PU	0.898			
IU	0.578	0.581	**1.000**		IU	0.604	0.613		
eBANK	0.792	0.777	0.569	**0.898**	eBANK	0.858	0.851	0.584	

*PEU, perceived ease of use; PU, perceived usefulness; IU, intention to use; eBANK, e-banking usage; AVE, average variance extracted. Fornell–Larcker criterion: diagonal elements (bold) are the square root of the variance shared between the constructs and their measures (average variance extracted). Off-diagonal elements should be larger than off-diagonal elements.*

To evaluate the validity of the attitude toward using e-banking, the variance inflation factor (VIF) is used to assess the lack of collinearity problems by the indicators (VIF < 5) (see Table 5). Finally, for the significance value of the weights, ATT4 and ATT6 are not significant. However, according to Hair et al. (2019), since there are no collinearity problems and the loads are greater than 0.5, these indicators are not deleted.

**TABLE 5:** Measurement model: model composite Mode B.

					Weights bootstrapping		Collinearity statistic

Constructs	Loading	Weights	*t*	Sig	5%	95%	VIF
Attitude toward using e-banking (Mode B)							
ATT1	0.879	0.524**	2.787	0.003	0.175	0.794	2.040
ATT2	0.864	0.317*	1.814	0.035	0.003	0.583	3.535
ATT3	0.541	0.237*	1.844	0.033	−0.455	−0.035	1.927
ATT4	0.795	0.065^(*ns*)^	0.414	0.339	0.166	0.352	3.154
ATT5	0.856	0.298*	1.839	0.033	0.010	0.548	4.002
ATT6	0.764	0.113^(*ns*)^	0.816	0.207	0.117	0.345	2.719

*n* = 5,000 subsample: **p < 0.01; *p < 0.05; ns, non-significant (one-tailed *t* Student).

### Structural Model

After checking the reliability and validity of the measurement model, the proposed structural model is examined. To do this, the explanatory capacity of the model is evaluated using *R*^2^ (Hair et al., 2019). The *R*^2^ values are 0.657 for perceived usefulness, 0.530 for attitude toward using e-banking, 0.462 for intention to use, and 0.324 for e-banking usage. After performing an analysis of the variance decomposition, the findings confirm that, of the 53% of the explained variance of attitude toward using e-banking, 29.1% is due to perceived ease of use and 23.9% to perceived usefulness. Similarly, of the 46.2% of explained variance of intention to use, 14.4% is due to perceived usefulness, and 31.8% is due to attitude toward using e-banking. Even though these results confirm significant relationships, the influence of consumers’ attitudes toward the intention to use e-banking is greater than the contribution of the perceived usefulness.

On the other hand, the path coefficients and their significance are evaluated to describe the significance of the structural relationships proposed in the model (see Table 6). Perceived ease of use appears to be positive and significant, at 99.9% in perceived usefulness. Thus, H1 is supported, being the most solid association of the model (β = 0.811). As proposed in H2 and H3, perceived ease of use and perceived usefulness are positively associated with the attitude toward using e-banking (β = 0.417 and 0.348, respectively). Similarly, perceived usefulness has a significant influence on the intention to use of e-banking, also confirming H4 (β = 0.248). Also, attitude toward using e-banking, in general, has a significant and positive effect on the intention to use e-banking. Thus, H5 is established (β = 0.485). Finally, the intention to use has a significant influence on e-banking usage. Therefore, H6 is also confirmed (β = 0.569) (Hair et al., 2019). Thus, hypotheses H1, H2, H3, H4, H5, and H6 are accepted by the percentile method.

**TABLE 6:** Structural model evaluation.

	β	*T*	Confidence interval (95%)	VARIANCE EXPLAINED (R^2^)		PREDICTIVE RELEVANCE (*Q*^2^)	Effect size (*f*^2^)	VIF
				*R*^2^	Adjusted *R*^2^			
PEUPU	0.811***	1.267	(0.744; 0.868) Sig.	0.657	0.654	0.539	1.915	1.000
PEUATT	0.417**	2.372	(0.128; 0.702) Sig.				0.127	2.915
PUATT	0.348*	1.687	(0.011; 0.684) Sig.	0.530	0.521	0.307	0.088	2.915
PUIU	0.248**	2.663	(0.060; 0.365) Sig.				0.061	1.888
ATTIU	0.485***	4.866	(0.350; 0.678) Sig.	0.462	0.452	0.390	0.232	1.888
IUeBANK	0.569***	9.733	(0.475; 0.667) Sig.	0.324	0.317	0.243	0.479	1.000

*n* = 5,000 subsample: ***p < 0.001; **p < 0.01; *p < 0.05 (one-tailed *t* Student). PEU, perceived ease of use; PU, perceived usefulness; ATT, attitude toward using e-banking; IU, intention to use; eBANK, e-banking usage.

After evaluating and confirming the proposed model, the effect size is evaluated (Hair et al., 2019). Thus, the results show that (see Table 6), the perceived ease of use has a large effect size on perceived usefulness (*f*^2^ = 1.915). Likewise, the intention to use has a significant and large effect size on e-banking usage (*f*^2^ = 0.479). Finally, the model’s predictive relevance is analyzed. In this case, Stone–Geisser’s *Q*^2^ shows that the scores are higher than naught (see Table 6).

**FIGURE 2:**
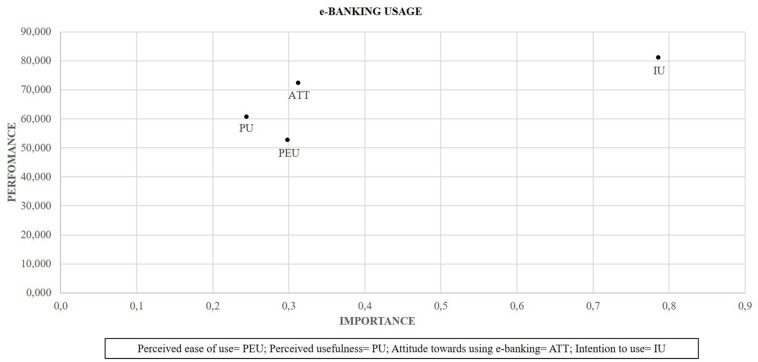
Importance-performance map analysis (IPMA) for e-banking usage.

To improve these results, the IPMA is used. The IPMA expands the reported PLS-SEM results for path coefficient estimates by adding a dimension to the analysis that considers the mean values of the latent variable scores (Ringle and Sarstedt, 2016). In this case, the IPMA for e-banking users (see Figure 2) shows that intention to use is observed to be the most critical factor in determining e-banking usage. An increase of one point in the performance of intention to use by a total effect of 0.786. Attitude toward using e-banking has higher importance on e-banking usage but lower than the intention to use. Similarly, the attitude has a lower performance than the intention to use. The perceived ease of use is the factor with the lowest performance. Finally, perceived usefulness has the lowest importance in determining e-banking usage (see Figure 2).

## Discussion

This study developed a research framework to understand the factors that contribute to e-banking usage and to benefit business strategies based on the co-creation of consumer value. The model provides a comprehensive view of the main factors influencing e-banking intentions and the elements that should be considered to increase usage.

The results obtained concerning the application of TAM in the context of e-banking confirm the presence of significant relationships among perceived ease of use and perceived usefulness by the customer, being the most real relationship of the proposed model. Similarly, the relationships between perceived usefulness and attitude toward using e-banking, perceived ease of use and attitude toward using e-banking are also contrasted. However, the importance that perceived ease of use acquires in the attitude toward using e-banking is slightly higher than the influence that perceived usefulness has on this variable. Similarly, the relationships between perceived usefulness and intention to use, and attitude toward using e-banking and intention to use are also contrasted with previous studies, such as Salimon et al. (2017), Zhang et al. (2018), and Malaquias and Hwang (2019). Nevertheless, the results of the analysis of variance decomposition indicate that attitude toward using e-banking has relatively greater importance in intention to use compared to perceived usefulness. Therefore, an essential contribution of this research is the determination of attitude as a critical element in the determination of e-banking use intention. These results suggest that when e-banking users have a positive attitude toward using e-banking, it translates into a greater intention to use e-banking. Finally, the relationship between intention to use and e-banking usage is also verified, being the second strongest relationship of the model. In this sense, the results obtained by the IPMA analysis indicate that the intention to use is the variable with the highest performance and the greatest importance in determining the adoption of e-banking. However, the perception of ease of use, despite the great importance in determining the use of e-banking, is the variable with the lowest performance in the proposed model.

These findings offer important implications for banks and financial institutions. The techniques and results of this study allow banks to identify possible deficiencies and apply improvements to establish greater interaction with their clients. Also, this study offers bank managers new tools that encourage co-creation through e-banking services, helping to achieve a competitive advantage.

Based on the results obtained, bank managers should pay special attention to the perceived ease of use and perceived usefulness of their e-services, since they contribute significantly to the adoption of e-banking by consumers. Perceived ease of use of e-banking services is one of the most relevant factors in the adoption of e-banking by consumers. However, the IPMA indicates that it is the factor with the lowest performance. As a consequence, banks can improve the usability and simplicity of their e-services and the performance of a banking transaction to facilitate and increase the e-banking usage. Likewise, customer service can be provided to guide and help the efficient use of these applications. Specifically, some authors such as Mostafa (2020) recommend the use of chatbot to facilitate the use of e-banking and co-create. Concurrently, the findings have shown the great importance of attitude in generating intention to use e-banking by consumers. Therefore, banks should encourage this attitude in consumers through the ease of use and usefulness provided by e-services.

By and large, as technology and smartphone advance, consumers will continue to seek out more personalized and utilitarian services for their banking operations. Therefore, e-banking should be secure, and easy to learn and use. For this reason, providing reliable, user-friendly, and useful e-services are a crucial element in the interactions between consumer adoption of e-banking.

### Limitations and Further Research

This study has some limitations that need to be addressed. The first limitation is the geographical location of the sample and the size of the sample. Future studies should incorporate a more significant number of online banking users covering a wider geographical area. Similarly, this study can increase the number of respondents between 34 and 53-year-old. Secondly, this study has not considered the moderating role of gender and age as socio-demographic variables. Previous authors, such as Natarajan et al. (2018), consider age as a great relevance in studies of acceptance of mobile applications. Further research may assess the moderating role of this variable in the proposed model. Thirdly, this model is based exclusively on functional characteristics of technology adoption, such as perceived ease of use and perceived usefulness. In the area of e-banking, authors such as Zhang et al. (2018) highlight other types of more emotional factors for the study of the adoption of e-banking services such as enjoyment or trust. Likewise, Singh and Srivastava (2020) highlight the perceived security in the factors of adoption of e-banking. Thus, a future proposal could include a combination of functional and emotional elements in e-banking environments. Finally, further research could incorporate external variables associated with value co-creation, such as the confidence in the bank. Some studies, such as Mostafa (2020), suggest that consumer confidence in the bank can intensify the positive effect of the attitude toward e-banking. If customers believe that their bank is honest and professional, their positive attitude toward the use of e-banking will result in a disposition to co-create value with the bank by sharing information or providing feedback.

## Data Availability Statement

The raw data supporting the conclusions of this article will be made available by the authors, without undue reservation.

## Author Contributions

All authors listed have made a substantial, direct and intellectual contribution to the work, and approved it for publication.

## Conflict of Interest

The authors declare that the research was conducted in the absence of any commercial or financial relationships that could be construed as a potential conflict of interest.
